# Outlier Removal in Model-Based Missing Value Imputation for Medical Datasets

**DOI:** 10.1155/2018/1817479

**Published:** 2018-02-04

**Authors:** Min-Wei Huang, Wei-Chao Lin, Chih-Fong Tsai

**Affiliations:** ^1^School of Medicine, China Medical University, Taichung, Taiwan; ^2^Department of Psychiatry, Chiayi Branch, Taichung Veterans General Hospital, Chiayi, Taiwan; ^3^Department of Information Management, Chang Gung University, Taoyuan, Taiwan; ^4^Department of Thoracic Surgery, Chang Gung Memorial Hospital, Linkou, Taoyuan, Taiwan; ^5^Department of Information Management, National Central University, Zhongli, Taoyuan, Taiwan

## Abstract

Many real-world medical datasets contain some proportion of missing (attribute) values. In general, missing value imputation can be performed to solve this problem, which is to provide estimations for the missing values by a reasoning process based on the (complete) observed data. However, if the observed data contain some noisy information or outliers, the estimations of the missing values may not be reliable or may even be quite different from the real values. The aim of this paper is to examine whether a combination of instance selection from the observed data and missing value imputation offers better performance than performing missing value imputation alone. In particular, three instance selection algorithms, DROP3, GA, and IB3, and three imputation algorithms, KNNI, MLP, and SVM, are used in order to find out the best combination. The experimental results show that that performing instance selection can have a positive impact on missing value imputation over the numerical data type of medical datasets, and specific combinations of instance selection and imputation methods can improve the imputation results over the mixed data type of medical datasets. However, instance selection does not have a definitely positive impact on the imputation result for categorical medical datasets.

## 1. Introduction

The first step in the data mining or knowledge discovery in databases (KDD) process is to collect a certain amount of data for a specific defined problem. However, in practice, it is usually the case that the medical dataset collected for later data mining steps is not complete due to problems such as manual data entry procedures, incorrect measurements, and equipment errors. As a result, the collected datasets generally contain some missing (attribute) values or missing data [9, 21].

For many data mining algorithms, it is not possible to develop learning models when used over incomplete medical datasets. Despite the fact that some algorithms, such as decision trees, can handle incomplete datasets without any preprocessing support [24], the final analysis or mining results can be greatly affected by the incomplete datasets. In other words, the prediction performance of the constructed model trained by an incomplete dataset is questionable.

There are two types of solutions used to solve the problem of missing values or incomplete datasets. The first solution, which is the simplest and most straightforward solution, is based on case deletion. With the case deletion approach, the (incomplete) data having missing values are discarded directly. However, this method is generally appropriate only when the chosen dataset contains a very small amount of missing data, for example, 5% missing rate. The second solution is based on missing value imputation. It can provide estimations for missing values by reasoning from the observed data (i.e., complete data) [13, 14, 20].

In the literature, the efficacy of some different missing value imputation algorithms used with different kinds of datasets containing various missing data rates has been compared. The experimental results have shown that missing value imputation is a better choice than case deletion when the incomplete datasets contain a certain amount of missing values. Model-based missing value imputation algorithms based on machine learning techniques, such as *k*-nearest neighbor, multilayer perceptron neural networks, and support vector machines, have recently lately been widely considered [14, 16, 21].

Since all of the model-based imputation algorithms require observed data without missing values in the incomplete dataset, as the training set to provide estimations of the missing values, the imputation results are directly affected by the observed data. From the view point of instance selection [12, 17], a given training set generally contains noisy data or outliers that can degrade the final performance of a learning model. The aim of instance selection is to filter out unrepresentative data from a given training set, and a learning model trained by the reduced training set is likely to perform better than the one trained by the original training set.

In other words, from the instance selection perspective, there would be some noisy data that exist in the observed dataset for missing value imputation. As a result, it is important to examine the performances of performing instance selection over the observed dataset before missing value imputation. In Tsai and Chang [22], different priorities of combining instance selection and imputation algorithms over various domain datasets were studied. Although they conclude that performing instance selection first and imputation second is the better combination process, they only use one specific instance selection algorithm combined with one specific imputation method for the experiments.

Therefore, the research objective of this paper is to examine whether methods combining instance selection and missing value imputation can outperform those using missing value imputation alone for incomplete medical datasets. The combination process is discussed below. Given an incomplete dataset, which contains complete data without missing values and incomplete data having missing values, the complete data for the imputation algorithms are selected by the instance selection process, and then missing value imputation is performed over the reduced set of complete data. For the medical domain classification problem, we aim to demonstrate that through different instance selection algorithms, the new imputation results by different imputation models can make the learning classifier performs better than the one using the original imputation results obtained without performing instance selection.

The contribution of this research is twofold. For missing value imputation, we show that using all of the observed data to produce the estimations to replace the missing values based on the baseline approach may not be the best imputation solution. That is, the quality of the observed data should be carefully considered. On the other hand, for instance selection, we demonstrate that it can be combined with the imputation process for incomplete medical datasets, which has never been done before.

In our experimental setup, three different instance selection algorithms and three different model-based imputation algorithms are combined interchangeably in order to find the best combination for the incomplete medical datasets. The real-world medical datasets can contain categorical (i.e., discrete), numerical (i.e., continuous), or both types of data. Here, three types of datasets with different missing rates ranging from 10% to 50% are used to assess the imputation performance.

The rest of this paper is organized as follows.  Section 2 overviews related literature including the missingness mechanisms, missing value imputation, and instance selection.  Section 3 describes the two imputation processes that are examined in this paper, which are the baseline imputation process and the process of combining instance selection and missing value imputation.  Section 4 presents the experimental setup and results. Finally, Section 5 concludes the paper.

## 2. Literature Review

### 2.1. The Missingness Mechanisms

Missing data randomness can be divided into three categories, namely, missing completely at random (MCAR), missing at random (MAR), and not missing at random (NMAR) [19].

MCAR is the highest level of randomness. Let *X* be the random attribute. If *P*(*X*∣^*x*^ missing) = *P*(*X*∣^*x*^ observed), then the distribution of *X* is not affected by missing values. Therefore, MCAR refers to data where the missingness mechanism does not depend on the attribute of interest, or any other attribute, which is observed in the data. In other words, it occurs when the probability of an instance (case) having a missing value for an attribute does not depend on either the known values or the missing data. Any missing data treatment method can be applied at this level of randomness without risk of introducing bias into the data.

On the other hand, for MAR, let *X* be the random attribute, and let *Z* be a set of predictor attributes. If *P*(*X*∣^*x*^ missing, *Z*) = *P*(*X*∣^*x*^ observed, *Z*), then the distribution of *X* is not affected by missing values for *X*∈*Z*. In other words, MAR occurs when the probability of an instance having a missing value for an attribute may depend on the value of that attribute.

NMAR occurs when the probability of an instance having a missing value for an attribute may depend on the value of that attribute. This is the most difficult condition to model. However, in practice, it is difficult to judge the missing data mechanism, as the values for the missing data are unknown.

### 2.2. Missing Value Imputation

Missing value imputation can be regarded as a pattern classification task. In pattern classification, each data sample is represented by a *d*-dimensional feature vector where *d* is the number of features or attributes. In addition, each feature vector usually belongs to one of *c* classes or categories. To develop a classifier, a given training set, composed of a number of training data, is used to train the chosen classification technique. For classification, a given testing set composed of a number of testing data having the same number of features that are unknown or new to the classifier, and the classifier, are used to classify each testing data sample into one of the learned *c* classes.

For missing value imputation, the given incomplete *d*-dimensional dataset can be divided into complete and incomplete subsets, to be used as the training and testing sets, respectively. For example, when its *k*th attribute of the *i*th incomplete data sample is missing (where *k* ∈ *d*), the *k*th attribute of the training data is used as the final classification output, and the other *d*-1 attributes, with the exception of the *k*th one, are used as the input features. The classifier is trained to classify the incomplete data having the *k*th missing attribute values [13].

The various missing value imputation methods can be classified into statistical and machine learning methods [5, 13]. Different imputation methods have been compared in the literature (e.g., [1, 5, 11]), and some novel imputation methods have been proposed in recent studies (e.g., [26, 27]).

In particular, *k*-nearest neighbor imputation (KNNI) [10] is one of the most popular approaches. It is based on the *k*-nearest neighbor classification principle where missing values are imputed using values calculated from the *k*-nearest neighbors. An important parameter for the KNNI method is the value of *k*, which is typically set to 1, but is sensitive to outliers. According to Jonsson and Wohlin [15], the performance is relatively unaffected by the value of *k* while Batista and Monard [5] report that *k* = 10 for large datasets.

For more detailed information about the other imputation methods, please refer to de Leeuw [8] and Garcia-Laencina et al. [13].

### 2.3. Instance Selection

The aim of instance selection is to filter out some noisy or unrepresentative data from a given (training) dataset. In practice, the collected data may not all be equally informative, and some data points are considered noisy points or outliers. Using the original dataset without excluding the outliers could lead to significant degradation in degradation [2], but performing instance selection is likely to increase generalization accuracy and the dataset size can also be reduced [25].

Instance selection can be defined as follows. Let *X*_i_ be an instance where *X*_i_ = (*X*_i1_, *X*_i2_,…, *X*_im_, *X*_ic_), meaning that *X*_i_ is represented by *m*-dimensional features and *X*_i_ belongs to class *c* given by *X*_ic_. Then, assume that there is a target set TA that consists of *M* instances, which is used for instance selection. Consequently, a subset of selected samples *S* is produced, where *S*⊆TA. Given a testing set TS, we can classify a new pattern *T* from TS over the instances of *S* and TA. If the instance selection algorithm has been chosen appropriately, the classifier performance trained by *S* should be better than that of TA.

A number of related studies proposing instance selection methods for obtaining better mining quality appear in the literature. Recently, Garcia et al. [12] compared fifty related instance selection algorithms over various datasets in terms of classification accuracy. They divided related algorithms into three types of techniques, which are edition, condensation, and hybrid methods. Generally speaking, edition methods aim to remove noisy data samples from a given (training) set in order to increase classifier accuracy. Condensation methods aim to remove redundant data samples where the classifier's performance trained by the reduced training set will not be affected. Hybrid methods focus on searching for a small subset by simultaneously eliminating both noisy and redundant data samples.

Although there is no exact winner for all of the problem datasets, they found that, on average, hybrid methods, such as genetic algorithms [6], IB3 [3], and DROP3 [25], are able to provide the largest data reduction rates and can make the trained classifiers outperform the ones without instance selection. In addition, since there is no generally agreed definition of outliers for different domain problems, the determination of outliers is based on the chosen instance selection method to filter out unrepresentative data samples from a given dataset.

## 3. The Two Imputation Processes

### 3.1. The Baseline Imputation Process

The baseline imputation process is described below. Given a dataset *D* with some missing values where each data sample is composed of a number of attributes and their associated class labels, the data with and without missing values can be denoted as complete (*D*_complete_) and incomplete subsets (*D*_incomplete_), where *D* ∈ *D*_complete_ + *D*_incomplete_.

To impute the *i*th missing attribute of the *j*th data sample in *D*_incomplete_, the *i*th attribute of *D*_complete_ is used as the output class for classification or prediction, and the other attributes, except for the original output class of *D*, are used as the input attributes (or variables). The resultant training set for estimating the *i*th missing attribute in *D*_incomplete_ is generated, while the data samples having the *i*th missing attribute in *D*_incomplete_ are used as the testing data.

In this paper, three different model-based imputation methods based on supervised learning techniques are considered for comparison: the KNNI (*k* = 1), multilayer perceptron (MLP) (the parameters of MLP are based on the default values of the Weka software), and support vector machine (SVM) (the parameters of SVM are based on the default values of LIBSVM [7]). The reason of choosing KNNI and MLP is because Garcia-Laencina et al. [13] compared KNNI, MLP, SOM (self-organizing map), and EM (expectation maximization) for missing value imputation and they found that KNNI and MLP perform similar and can provide better performances than SOM and EM. Note that for the numerical data type of datasets, support vector regression is used. The imputation result is based on the output of each method (or classifier) over the testing dataset.

After the imputation process is completed, which means that the original dataset *D* is imputed, denoted as *D*′, as a *pseudo-complete* dataset, then, *D*′ is used as the training set and *T* for the testing set to train and test the SVM classifier, respectively. The final classification result is regarded as the evaluation metric and is used to examine the imputation performance of these three imputation methods.

### 3.2. The Process of Combining Instance Selection and Imputation

Differing from the baseline imputation process, in this process, instance selection is performed first, and then the output is used for missing value imputation. The first step is to choose a specific instance selection method for removing some of the noisy data from the complete subset *D*_complete_. The resultant reduced subset, denoted as *D*_complete_reduced_, is produced. Next, *D*_complete_reduced_ and *D*_incomplete_ are combined (now denoted by *D*_reduced_) for missing value imputation by the three chosen imputation methods (i.e., KNNI, MLP, and SVM) individually. Note that the number of data samples in *D*_reduced_ is smaller than in *D* (and *D*′). Finally, after performing imputation, the reduced dataset *D*_reduced_ becomes a *pseudo-complete* reduced dataset, denoted as *D*'_reduced_.

In particular, during the instance selection step, three instance selection methods are employed for comparison, namely, IB3, DROP, and genetic algorithms (GA). They have been widely used as the baseline instance selection algorithms in related studies [18, 23]. There are nine different combinations of instance selection and imputation methods for each dataset: IB3/DROP3/GA + KNNI, IB3/DROP3/GA + MLP, and IB3/DROP3/GA + SVM.

Similar to the final step of the baseline imputation process, the SVM classifier is trained and tested by *D*'_reduced_ and *T*, respectively. Consequently, the classification accuracy of SVM trained by *D*′ and *D*'_reduced_ over the testing set *T* and the results are compared to examine the instance selection effect.

## 4. Experiments

### 4.1. Experimental Setup

Three different attribute types of medical datasets are chosen from the UCI Machine Learning Repository (http://archive.ics.uci.edu/ml/), comprising categorical, numerical, and mixed attribute types of data, containing 4, 5, and 6 datasets, respectively. Moreover, each type of dataset contains different numbers of attributes, samples, and classes, which are helpful in determining the instance selection effect of using different types of datasets with different missing rates on the final classification accuracy.  Table 1 lists the basic information for these datasets.

Each medical dataset is divided into a 90% training (*D*) and 10% testing (*T*) set based on the 10-fold cross-validation strategy. In addition, to examine the effect of performing instance selection on missing value imputation, five different missing rates for each dataset *D*, which are 10%, 20%, 30%, 40%, and 50% at 10% intervals, are simulated. Particularly, they are simulated by the MCAR (missing completely at random) mechanism, which is the most widely considered in related studies because MCAR is easy to be empirically tested [4]. Since larger missing rates by MCAR may cause each data sample containing at least one or more missing values, that is, there is no complete data sample in *D* for the imputation model, the criterion of simulating different missing rates is that at least five complete data samples containing no missing values should exist in *D*.

Moreover, in order to reduce the likelihood of obtaining biased results by randomly introducing missing values, each missing rate calculation is performed 10 times over each *D*. As a result, ten incomplete datasets are generated from each *D* with one specific missing rate. Then, the two imputation processes are executed over the incomplete datasets for performance comparison.

### 4.2. Results on Categorical Medical Datasets

**Figure 1:**
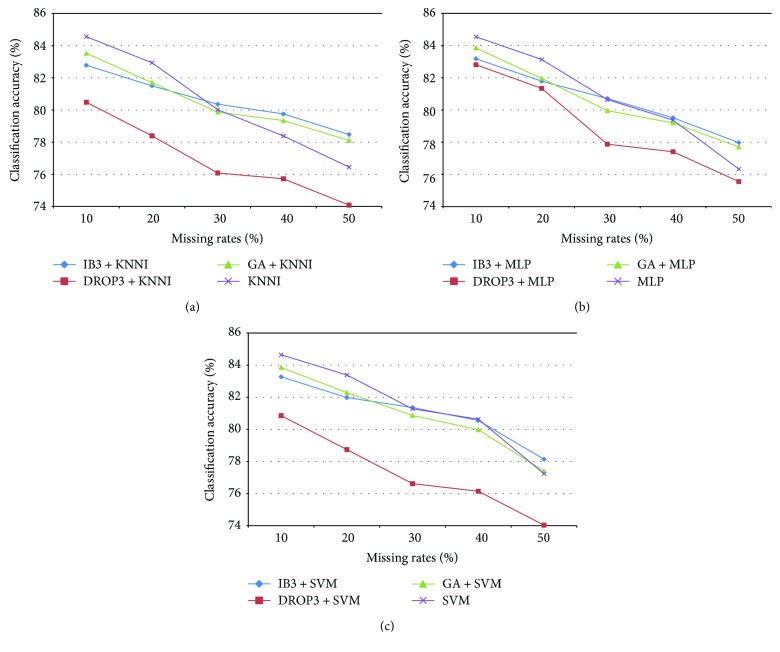
Classification results of imputation and instance selection combined with imputation over the categorical medical datasets.

Figure 1 shows the average classification accuracy of SVM obtained using different imputation processes over the categorical medical datasets with different missing rates. We can see that there is a gradual degradation in the classification accuracy as the missing rates increase. This indicates that datasets with more missing values (i.e., larger missing rates) limit the complete data samples used as the training set for imputation, which is likely to make the classifier provide lower classification accuracy. This finding can be applied to two types of datasets.

Specifically, lowest rates of classification accuracy are obtained with the SVM classifier, combining DROP3 for instance selection with the three imputation methods. In particular, performing instance selection by IB3 and GA can provide significantly better imputation results than using DROP3 (*p* < 0.01).

On the other hand, we found that when the missing rates fall below 30% (i.e., 10% and 20%), there is no need to consider performing instance selection before imputation over categorical datasets. This is because the SVM classifier based on the baseline imputation methods significantly outperforms the one based on the combined methods (*p* < 0.01). In this case, the SVM imputation method performs the best and MLP the second best.

However, it is interesting that when the missing rates are larger than 30%, performance is slightly better when instance selection is combined with imputation than with the baseline imputation methods. In particular, combining the IB3 instance selection method with the imputation methods (i.e., KNNI, MLP, and SVM) significantly performs better than the baseline imputation methods (*p* < 0.05). Therefore, performing instance selection could have some positive impact on missing value imputation over categorical datasets with the missing rates larger than 30%.

### 4.3. Results on Numerical Medical Datasets

**Figure 2:**
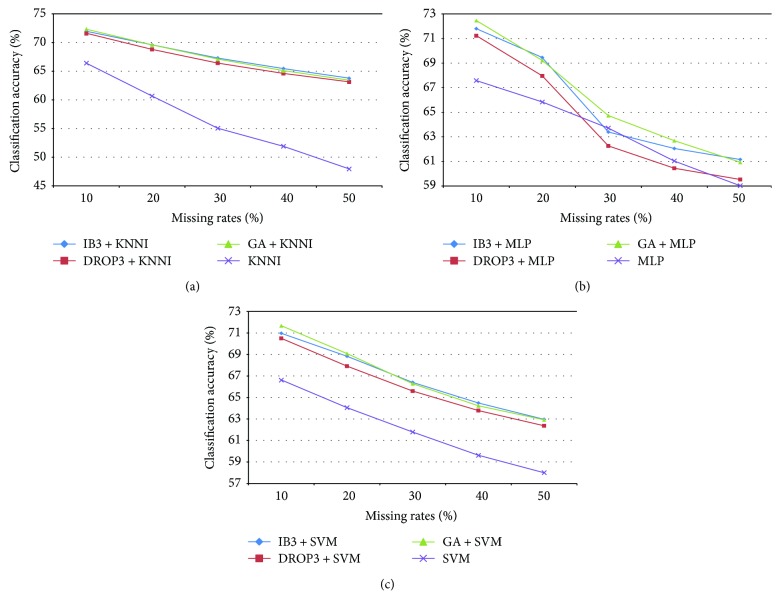
Classification results of imputation and instance selection combined with imputation over the numerical medical datasets.

Figure 2 shows the average classification accuracy of SVM produced by the different imputation processes over the numerical medical datasets with different missing rates. The results indicate that, in most cases, the combination of instance selection and imputation performs better than the baseline imputation methods (i.e., with different missing rates).

Although the differences in performance between most of the combinations are very small, that is, below 2% of classification accuracy, we still can find out that the best combination is based on GA + MLP for the 10% missing rate and IB3 + KNNI for the 20% to 50% missing rates, which significantly outperform the other combinations and the baseline imputation methods (*p* < 0.01).

In contrast, comparison of the performance degradation with missing rates from 10% to 50% shows that the most stable classification performance is obtained with the SVM classifier based on IB3 + SVM. Specifically, the performance degradation of SVM based on IB3 + SVM with 10% to 50% missing rates is 8%, whereas DROP3 + SVM is the second best (i.e., 8.13%) and IB3 + KNNI is the third best (i.e., 8.18%).

These results demonstrate that performing instance selection has a positive impact on missing value imputation over most numerical datasets. Furthermore, the choice of instance selection method does not significantly affect the imputation results.

### 4.4. Results on Mixed Medical Datasets

**Figure 3:**
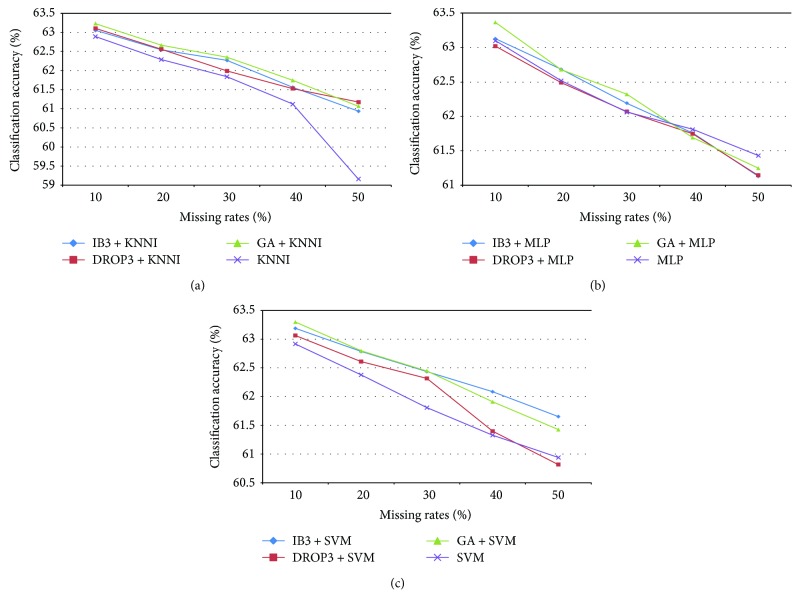
Classification results of imputation and instance selection combined with imputation over the mixed medical datasets.

Figure 3 shows the average classification accuracy of the SVM obtained by different imputation processes over mixed medical datasets with different missing rates. We can see that performing instance selection can improve the imputation results, except for the MLP imputation method combination. In addition, it should be noted that the smallest performance improvement is obtained using DROP3 for instance selection when compared with IB3 and GA. The best combination is based on GA + MLP for 10% missing rate and IB3 + SVM for 20% to 50% missing rates, which significantly outperform the other combinations and the baseline imputation methods (*p* < 0.01). However, the performance differences between them are very small, that is, below 1% of classification accuracy.

Therefore, it can be concluded that with this type of datasets, performing instance selection may have a positive impact on the imputation results, if the instance selection and imputation methods are carefully chosen. The classifier performs better when using IB3 or GA for instance selection and KNNI or SVM for missing value imputation than performing the imputation step alone.

### 4.5. Further Comparisons

The best imputation and combined methods that significantly outperform the other methods (*p* < 0.05) used over each medical dataset with different missing rates are listed in Table 2. There is no exact winner for different medical domain datasets with different missing rates. The findings obtained based on the dataset characteristics, such as the dimensionalities (i.e., number of attributes), the dataset sizes (i.e., number of instances), and the number of classes, are discussed below.

In categorical medical datasets, when their dimensionalities are low (e.g., lower than 22) and they belong to two-class classification problems, such as the SPECT dataset, meaning that when the dataset contains a relatively low complex problem, it is sufficient to use the baseline imputation process for most of the different missing rates. However, when the categorical medical datasets contain very high dimensionalities, such as Promoters, performing instance selection may improve the imputation result.

On the other hand, for most numerical datasets, performing instance selection to filter out some noisy data can improve the imputation result. Similar to the numerical datasets, better results can be obtained by combining instance selection and imputation over most of the mixed medical datasets. The exception is the Acute dataset, which contains a small number of data samples, attributes, and classes (i.e., 160, 6, and 2, resp.), so better performance is obtained with the baseline imputation process than the combined process.

In summary, it is difficult to conclude whether combining instance selection and imputation is the better choice by only looking at one specific dataset characteristic, such as numbers of attributes. The three dataset characteristics usually relate to each other for each specific domain problem dataset. In spite of this, these experimental results show that performing instance selection before missing value imputation is recommended for most of the cases.

## 5. Conclusion

The incomplete dataset problem is usually approached by missing value imputation. In the past, many different types of imputation algorithms have been studied. Model-based algorithms based on machine learning techniques have been applied recently. The imputation result is heavily dependent on the reasoning process used to process the observed data or training data, and the quality of the data that the imputation algorithms use to produce estimations to replace missing is an important issue.

In this paper, we focus on examining whether performing instance selection to filter out some noisy data from a given training medical dataset has a positive impact on the final imputation results. Specifically, the aim is to compare the classification performance obtained through the processes combining instance selection and imputation and the baseline imputation process. Three types of medical datasets including categorical, numerical, and mixed types of data are used. This allows us to identify the effect of performing instance selection on missing value imputation and understand when we should consider instance selection before imputation.

Three different instance selection methods and three model-based imputation algorithms are compared. Our experimental results show that performing instance selection first mostly improves the imputation result over these three types of medical data. In particular, we found that the negative impact is to consider instance selection before imputation when the dataset contains lower dimensionalities and numbers of classes. However, for numerical datasets, the combined instance selection and imputation process performs better than the baseline imputation process for most datasets with different missing rates. Finally, for mixed datasets, the instance selection effect is between that for categorical and numerical datasets, which means that combining instance selection and imputation could be a better choice if both kinds of algorithms are carefully chosen.

Several issues could be considered in future work. First, since we only focus on the missing completely at random (MCAR) mechanism, other mechanisms should be considered with different types of medical data to fully examine the instance selection effect. Second, as there is no generally agreed upon definition of outliers, different instance selection algorithms will usually filter out different data samples from the same dataset. We think that fusing multiple instance selection results by the union or intersection strategy may produce a better quality of the observed (training) data for missing value imputation. Third, similar to the second issue, the imputation result could be improved if multiple imputation results were combined by different imputation algorithms. Last but not the least, some real-world big medical datasets containing very large volumes of data samples with high dimensionalities should be used for further study in order to conclude whether performing instance selection has a positive impact on missing value imputation.

## Figures and Tables

**Table 1:** Dataset information.

Dataset	Number of instances	Number of attributes	Number of classes
*Categorical datasets*			
Lymphography	148	18	4
Nursery	12960	8	11
Promoters	106	58	2
SPECT	267	22	2
*Numerical datasets*			
Blood	748	5	2
Breast cancer	286	9	2
*E*. *coli*	336	8	8
Pima	768	8	2
Yeast	1484	8	10
*Mixed datasets*			
Abalone	4177	8	29
Acute	120	6	2
Contraceptive	1473	9	3
Liver_disorders	345	7	2
Statlog	270	13	2
Statlog_German	1000	20	2

**Table 2:** The best imputation process over each dataset.

Dataset	Missing rate				
	10%	20%	30%	40%	50%
*Categorical datasets*					
Lymphography	GA + SVM	IB3 + KNNI	DROP3 + SVM	IB3 + KNNI	DROP3 + KNNI
Nursery	KNNI	GA + MLP	IB3 + MLP	MLP	MLP
Promoters	DROP3 + MLP	IB3/DROP3 + KNNI	IB3/DROP3 + MLP	IB3/DROP3 + SVM	IB3/DROP3 + SVM
SPECT	KNNI	MLP	MLP	MLP	KNNI
*Numerical datasets*					
Blood	GA + KNNI	GA + MLP	GA + MLP	GA + KNNI	DROP3 + MLP
Breast cancer	IB3 + SVM	IB3 + MLP	IB3 + SVM	IB3 + SVM	IB3 + SVM
*E*. *coli*	IB3 + KNNI	IB3 + KNNI	IB3 + KNNI	IB3 + KNNI	IB3 + KNNI
Pima	IB3/DROP3/GA + KNNI/MLP/SVM	IB3 + MLP	IB3 + KNNI/MLP DROP3 + KNNI GA + MLP	IB3 + KNNI/MLP	IB3 + MLP
Yeast	IB3 + SVM	IB3 + KNNI	IB3 + SVM	IB3 + SVM	GA + SVM
*Mixed datasets*					
Abalone	IB3 + SVM	GA + MLP	GA + MLP	IB3 + SVM	GA + MLP
Acute	GA + SVM KNNI/MLP/SVM	MLP/SVM	KNNI/SVM	SVM	MLP
Contraceptive	KNNI	SVM	SVM	IB3 + SVM	MLP
Liver_disorders	IB3 + KNNI	IB3 + KNNI	IB3 + KNNI	IB3/GA + SVM	IB3 + KNNI
Statlog	IB3 + KNNI/MLP	IB3 + KNNI/MLP/SVM GA + MLP	GA + SVM	IB3 + SVM GA + MLP/SVM	IB3 + MLP
Statlog_German	IB3/DROP3/GA + KNNI/MLP/SVM	IB3/DROP3/GA + KNNI/MLP/SVM	IB3/DROP3/GA + KNNI/MLP/SVM	IB3/DROP3/GA + KNNI/MLP/SVM	IB3/DROP3/GA + KNNI/MLP/SVM
